# Case report: Bilateral eye injuries in members of one family due to a cluster munition in Ukraine

**DOI:** 10.3389/fmed.2023.1171954

**Published:** 2023-05-18

**Authors:** Katarzyna Nowomiejska, Katarzyna Adamczyk, Dariusz Haszcz, Nataliya Preys, Robert Rejdak

**Affiliations:** ^1^Chair and Department of General and Pediatric Ophthalmology, Medical University of Lublin, Lublin, Poland; ^2^Lviv Regional Hospital, Lviv, Ukraine

**Keywords:** bilateral multiple IOFBs, open- and closed-globe injuries, severe vision loss, combat injury, pediatric eye injuries

## Abstract

The aim of the study was to report the effects of surgical treatment of three victims of a cluster munition in Ukraine. A 32-year-old woman and her sons—6-year-old male twins—presented in Poland after 18 days of delay in treatment. All ocular injuries were bilateral. One of the boys presented with total retinal detachment and a post-traumatic cataract as well as corneal sutures in one eye and a post-traumatic cataract in the other eye. The other boy had already developed atrophy in one eye and a vitreous hemorrhage in the other eye. The woman suffered from bilateral post-traumatic cataract with multiple glass intraocular foreign bodies (IOFBs). The surgical treatment included cataract surgery with intraocular lens implantation in three eyes, removal of IOFBs in one eye, and enucleation of the atrophic eye with implantation of an ocular prosthesis preventing constriction of face tissues. The eye with retinal detachment underwent pars plana vitrectomy, and the vitreous hemorrhage resolved itself. Postoperatively, visual acuity improved significantly in four of six eyes. Only in the eye with an open-globe injury and persistent retinal detachment, the final visual acuity was still poor. In conclusion, cluster munition may lead to bilateral ocular trauma with IOFBs, open-and close-globe injuries, and severe vision loss if left untreated. Modern ophthalmic surgery leads to vision with IOL improvement and solving the eyes after severe combat injury.

## Introduction

Ocular trauma is still the most common cause of unilateral blindness worldwide (1). Each year, approximately 1.6 million people become blind due to injuries; additionally, 2.3 million people develop bilateral low vision for this reason, and almost 19 million people suffer from unilateral blindness or low vision. Ocular trauma can cause permanent visual or cosmetic defects in the affected individuals. The incidence of ocular injuries has increased during military actions in the past years. Children are the most-at-risk population and are often the most severely affected in military conflicts (2).

The war in Ukraine, which started on the 24th February 2022, triggered one of the largest and fastest refugee crises in Europe since the end of the second world war. The number of refugees who had crossed the Ukrainian-Polish border by 11 April 2022 exceeded 2.68 million. These were mainly women and children, so the priority was to take care of their basic physiological needs (3). The healthcare needs of the Ukrainian refugees were mostly related to injuries, poisoning, malnutrition, and their lack of proper care in the country of origin (4). Ukrainian citizens have been granted medical care in Poland, where national medical care is publicly funded (5).

Children, unlike the adult population, are merely subjected to the horrors of war due to the lack of independence and life experience. War experience and displacement have profound effects on children’s affective development and mental health (6). Eye injuries can lead to serious conditions, ranging from the loss of vision to the loss of globes (7, 8). Moreover, eye trauma can have implications on the development of a child’s face.

A cluster munition is a kind of bomb that scatters into smaller sub-munitions intended to kill or mutilate on impact (9), and it can lead to injuries to the head and face regions. The eyeball is extremely vulnerable to explosive injuries since it is an exposed and incompressible spherical organ full of liquid and rich in vascular networks, with fragile tissues and fine structures (10). Due to the complexity of explosive injury mechanisms, the difficulty of diagnosis and treatment increases in patients with ocular explosive injuries. A detailed description of ocular blast injuries is particularly important for timely and accurate treatment to minimize the visual disability rate (10).

The aim of the study is to present the surgical ophthalmological treatment applied to three members of a Ukrainian family—a female and her sons—all affected by a cluster munition in Ukraine.

### Case presentation

This is a descriptive case series study. Written informed consent was obtained from all the patients. A young female and her children, 6-year-old male twins, were affected by a cluster munition during their stay at home in Donetsk in Ukraine on the 11th March 2022. At first, they were treated in the hospital in Donetsk: one twin underwent suturing of the corneal and lid wounds in the right eye, and the second twin probably had suturing of eye rupture in one eye. Moreover, small foreign bodies were removed from the skin of the face of all these patients. The family crossed the Ukrainian-Polish border on 28th March. Between the 29th March and the 15th April 2022, they were treated surgically at the Chair and Department of General and Pediatric Ophthalmology of the Medical University of Lublin, Poland.

### Case 1

**Figure 1:**
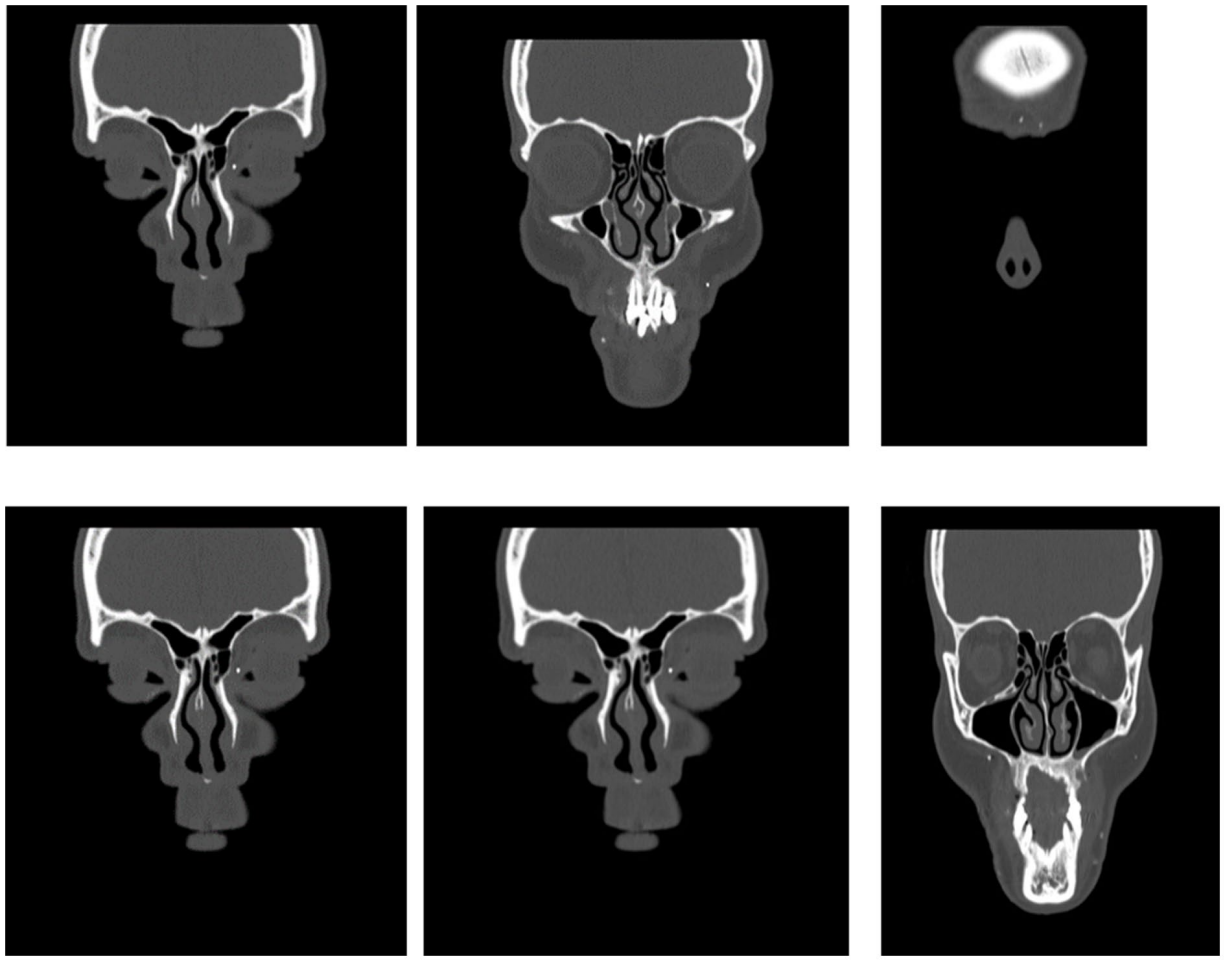
Preoperative computed tomography scan of the head of case 1. Multiply foreign bodies in the soft tissues of the face.

**Figure 2:**
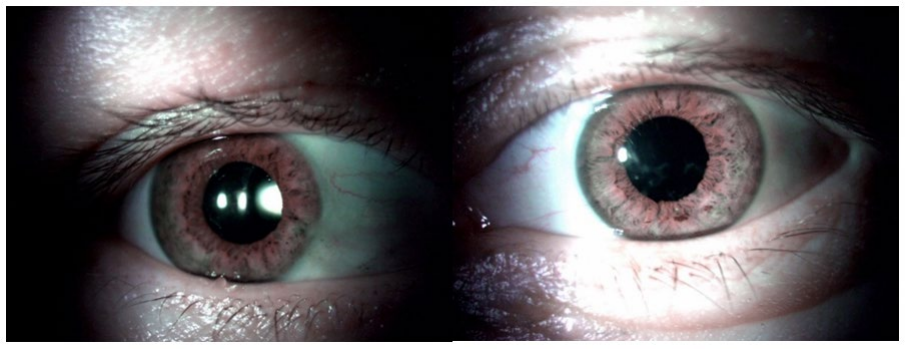
Anterior segment of both eyes of case 1 as seen postoperatively. **(A)** Right eye after the cataract surgery and intraocular lens implantation. **(B)** Left eye after the cataract surgery and intraocular lens implantation, corneal opacities paracentrally.

The 32-year-old woman presented with the visual acuity of counting fingers to 4 m in both eyes. She also had a broken leg and multiple foreign bodies in the skin of her face (Figure 1). A slit-lamp examination revealed bilateral traumatic cataract. The retina was attached in the ultrasonography. The patient underwent bilateral cataract surgery under peribulbar anesthesia with intraocular lens (IOL) implantation. During the surgery, multiple glass foreign bodies were found and removed from the crystalline lens of the left eye, anterior vitrectomy was performed, and posterior synechiae between the iris and the lens were removed. Postoperatively, after 9 months of the follow-up, visual acuity was 1.0 in both eyes with correction; however, in the left eye, corneal opacities are still present (Figure 2). The fundus was normal in both eyes. The injury in both eyes was classified as a close globe injury according to Birmingham Eye Trauma Terminology (BETT) (11).

### Case 2

**Figure 3:**
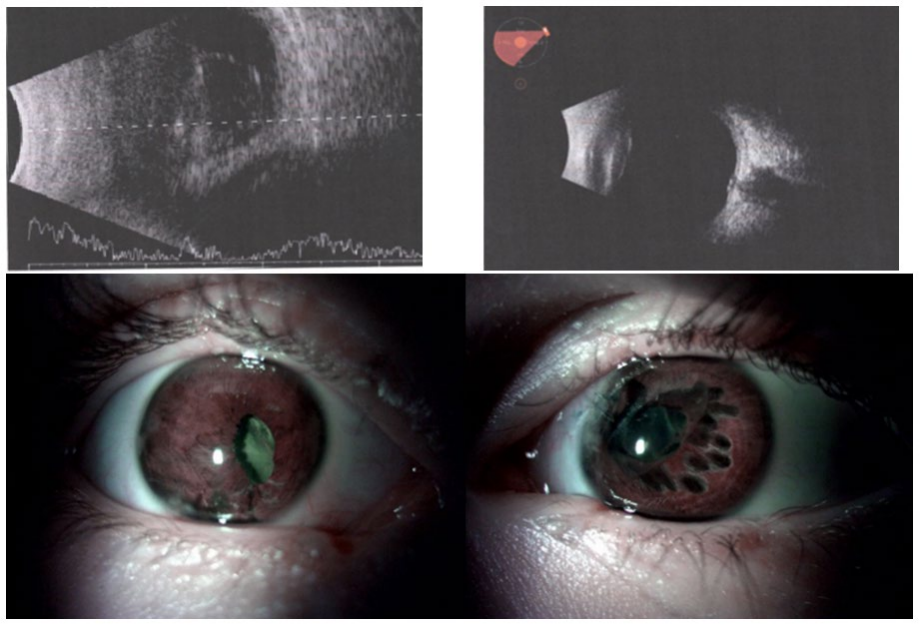
In the upper part, preoperative B-scans of two eyes of case 2. On the left, the right eye—retinal detachment; on the right, the left eye with attached retina and posttraumatic cataract. In the lower part, Anterior segment of both eyes of the case 2 as seen postoperatively. **(A)** Right eye after pars plana vitrectomy, cataract surgery, and intraocular lens implantation. **(B)** Left eye after post-traumatic cataract surgery and intraocular lens implantation. The holes seen in the iris are due to the cauterization performed to center the pupil.

A 6-year-old boy presented with an open globe injury (corneal laceration) in the right eye (zone 1 according to the Ocular Trauma Classification System) (12) and lid injury, primarily repaired with the corneal and lid sutures in Ukraine. He had also multiple foreign bodies in the skin of his face. Injuries of other organs were excluded. At the time of presentation for treatment in Poland, he had persistent total retinal detachment confirmed by B-scan ultrasonography and a traumatic cataract in the right eye. Visual acuity was light perception in the right eye. In the left eye, there was traumatic cataract, with the visual acuity of 0.1. Pars plana vitrectomy (PPV) combined with phacoemulsification and IOL implantation, removal of the corneal sutures, retinotomy, and 5,000 cSt silicone oil tamponade were performed under general anesthesia. In the left eye, there was a traumatic cataract treated with cataract extraction, posterior capsulorhesis, and IOL implantation, under general anesthesia. As the pupil was not well-centered, the cauterization of the iris was also performed in order to center the pupil. This intervention resulted in holes within the iris tissue (Figure 3). The postoperative visual acuity after 9 months corresponded to light perception in the right eye and 0.8 in the left eye. The injury in the right eye was classified as an open globe injury in the right eye and as a close globe injury according to BETT in the left eye (11).

### Case 3

**Figure 4:**
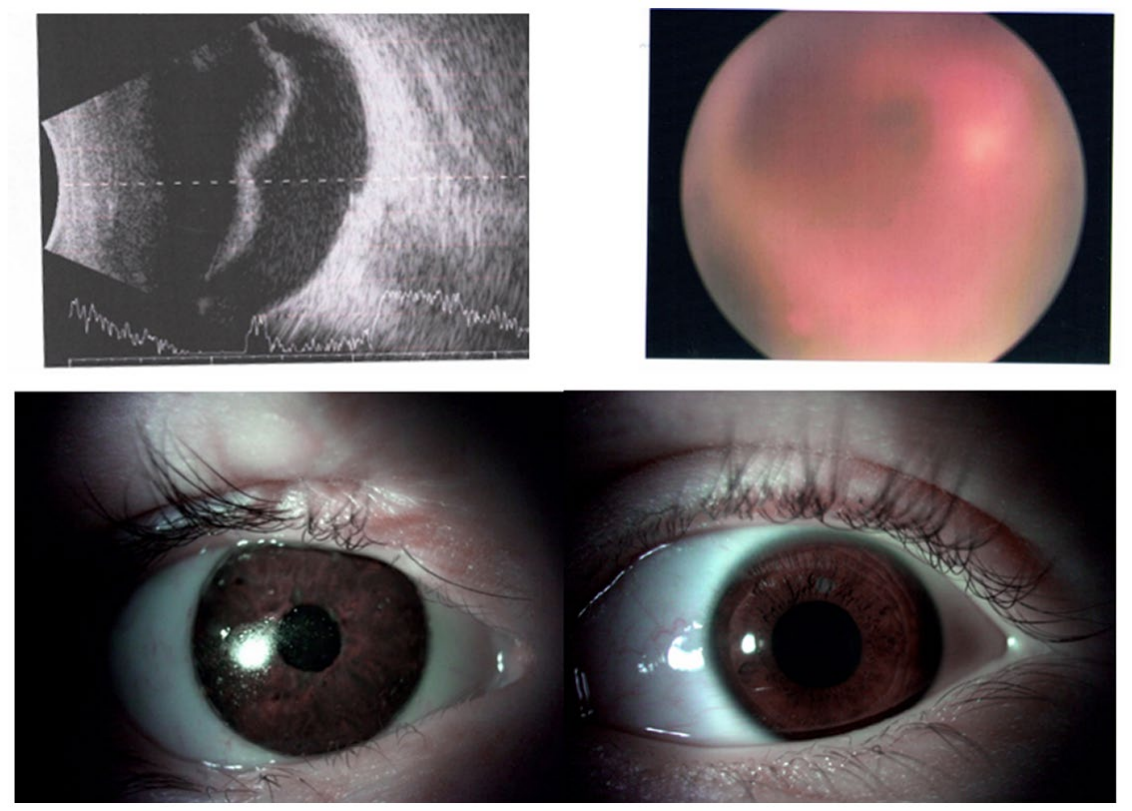
Upper side: On the left, preoperative ultrasonography B-scan of the left eye with the vitreous hemorrhage of case 3. On the right, preoperative fundus picture of the left eye with the vitreous hemorrhage. Lower side: Anterior segment of both eyes of case 3 as seen postoperatively. On the left, the right eye—an ocular prosthesis given after enucleation. On the right, the left eye—postoperative picture after traumatic cataract surgery and intraocular lens implantation.

The second 6-year-old boy presented with the phthisis bulbi in the right eye, probably after an eye globe rupture (11), and a vitreous hemorrhage in the left eye due to a closed-globe injury. He had also multiple foreign bodies on the skin of his face. Injuries of other organs were excluded. Vitreous hemorrhage in the left eye resolved after 7 weeks without treatment, and the visual acuity in the left eye improved from 0.1 to 1.0 without correction. After 3 months (119 days), the atrophic right eye globe was enucleated completely, and an orbital conformer was used, followed by an ocular prosthesis with a diameter of 16 mm to avoid constriction of tissues of the face (Figure 4).

All the clinical data of three patients (cases 1–3) are presented in Table 1.

**Table 1:** Clinical data of three presented cases including patient ID, classification and level of injury for every subject, diagnosis, visual acuity before and after treatment, timeline of treatment, and type of treatment.

Patient ID	Classification of injury	Diagnosis	Visual acuity (Snellen) with correction before treatment	Visual acuity (Snellen) with correction after treatment	Time from injury to surgical treatment	Treatment
Case 1 right eye	Close globe injury	Traumatic cataract	Counting fingers to 4 meters	1.0 with correction	20 days	Cataract surgery with intraocular lens implantation
Case 1 Left eye	Close globe injury	Traumatic cataract	Counting fingers to 4 meters	1.0	21 days	Cataract surgery with intraocular lens implantation and intralenticular foreign body removal
Case 2 right eye	Open globe injury	Corneal laceration (zone 1), traumatic cataract, retinal detachment	Light perception	Light perception	1 day 19 days	Primary sutures in Ukraine, Cataract surgery combined with intraocular lens implantation, pars plana vitrectomy with silicone oil tamponade
Case 2 left eye	Close globe injury	Traumatic cataract	0.1	0.8	26 days 145 days	Cataract surgery with intraocular lens implantation, cauterization of the iris
Case 3 right eye	Open globe injury	Globe rupture	Phthisis bulbi	Prosthesis bulbi	119 days	Enucleation with ocular prosthesis
Case 3 left eye	Close globe injury	Vitreous hemorrhage	0.1	1.0	49 days	Observation

## Discussion

In this study, we investigated the effects of surgical treatment of different types of eye trauma sustained by three members of one family affected by a cluster munition in Ukraine.

Eye injuries caused by bomb explosions are associated with severe ocular and systemic morbidity. Blast-associated injuries have already been demonstrated to result in poor functional outcomes despite surgical intervention because of the surgical complexities and extensive blunt ocular concussive damage (13). Eye injuries had previously been reported as a result of conflicts in Syria (14), Somalia (15), Pakistan (8, 16), and Iraq (17, 18). Based on that experience, it is already known that the most common causes of today’s combat ocular injuries are unconventional fragmentary munitions causing significant blast injuries (19). It has been concluded that the extreme severity of combat-related open-globe injuries leads to high rates of primary and retained IOFBs. IOFBs in combat-related ocular injury may have a reduced risk of endophthalmitis since they are usually propelled with high velocity from the explosion and may attain high temperatures, leading to self-sterilization. Predictably, visual outcomes are substantially associated with the depth of penetration of the IOFBs rather than with the timing of removal (20). This was also the case in the family described in this study. In both eyes of case 1, IOFBs were present in the crystalline lens and removed during cataract surgery. In spite of the delay of treatment of 18 days, there were no signs of endophthalmitis.

The delay of treatment of intralenticular IOFBs may also result in siderosis (21), however, not in this case, as IOFBs were non-metallic and consisted of glass. Glass IOFBs are often challenging to remove due to their polygonal shape, irregular borders, and smooth surfaces.

According to Sayer (22), there are different types of injuries that may be caused by cluster munitions. First, there is a shock wave due to pressure differences which can create injuries such as tympanic membrane rupture (called level I explosive injury). Second, there are fragments propelled by the explosive material due to kinetic energy (level II injury). These typically result in open-globe injuries and adnexal lacerations. Third, there is blast wind resulting in closed-globe injuries and orbital fractures (level III injury). Finally, there is intense heat from the explosive material, which can cause severe burns or high-pressure chemical reaction (level IV injury). In the case-series of six eyes, there were different types of eye injury, open-globe injuries in four eyes and close-globe injuries in two eyes. One eye had to be removed.

In a recent study by Zhang et al. (10), explosive eye injuries of 1,449 eyes in 1115 patients in China have been evaluated. Bilateral eye injuries were reported in 29.96% of cases. IOFBs resulted in 55.17% of open-globe injuries and contusion caused 60.22% of close-globe injuries. Nearly 9.59% of eyes were removed, and the final vision was ≤ 4/200 in 45.82% of patients. They concluded that explosion ocular trauma has complex mechanisms, with multiple eyes involved and poor prognosis. It has been already reported that the percentage of bilateral eye injuries is much higher in explosions (3.33–72.91%) (23–25) than in injuries of different origins (0–2.13%) (26, 27). In the present study, all patients had bilateral eye injuries.

In a study related to the war in Syria, 78 eyes were analyzed. A total of 44 eyes (56.4%) had displayed a traumatic cataract, 44 (56.4%) had retinal tears, 42 (53.8%) had vitreous hemorrhages, 18 (23%) had retinal detachment, 12 (15.3%) had endophthalmitis, and eight (10.2%) had hyphema. IOFBs consisted of metal in 62 eyes (79.4%), stone in eight eyes (10.2%), organic material in four eyes (5.1%), and glass in four eyes (5.1%). Approximately 86% of the eyes displayed initial visual acuity of 4/200 or worse. However, visual acuity improved in 64 eyes (82%) after surgical treatment (14). Victims of the civil war in Syria were mostly adult males; however, patients under 18 were present in 14% of the cases.

In a study conducted on victims of the war in Iraq, IOFBs were found in 166 eyes of 149 patients (18.6%). Most patients had a single IOFB (80.7%). The removal of IOFB was performed in 118 eyes (71.08%; an average of 31.67 days after the initial injury) with a delayed procedure occurring after primary closure and antibiotics owing to a lack of surgical capacity in Iraq and Afghanistan. Retinal detachment occurred in 48 eyes (28.92%) and proliferative vitreoretinopathy in 44 eyes (26.5%) (17). The traumatized eyes described in Iraq were the eyes of military soldiers, and patients were predominantly adult males.

In our case series, post-traumatic cataracts and vitreous hemorrhages were the prevailing ocular injuries found. Similar results were reported by Kalyachi et al. (15)—34 and 31%, respectively, and by Alam et al. (8)—30 and 38%, respectively.

We applied the standard surgical treatment performed in the case of anterior (cataract surgery) and posterior segment (PPV) injuries. Visual acuity improved significantly postoperatively in eyes with cataract and IOFBs and in eyes with vitreous hemorrhage without treatment. Eyes with retinal detachment of case 2 had finally very poor visual acuity due to delay of treatment with vitrectomy. The timing is especially important in regard to posterior segment eye injuries, and, in this case, the surgical intervention was conducted very late. It was delayed by more than 14 days, which can lead to proliferative vitreoretinopathy (PVR) and low visual acuity (28, 29). Retinal lesions, lens disruption, and vitreous hemorrhages are factors providing a medium for migrating cells to stimulate PVR. Delayed surgical treatment of posterior segment ocular trauma can also lead to the development of phthisis bulbi if not operated (30). One eye of case 3 was diagnosed as phthisis bulbi and was enucleated in order to prevent malformation of the face, and one eye of case 2 had delayed PPV and its functional results were poor. Retinal detachment due to an injury in children is associated with a worse visual prognosis (31, 32). Ocular trauma necessitating enucleation is less common but still not a rare injury. Anatomic reconstruction of the lost eye is essential to prevent or minimize the multitude of problems common to the post-traumatic anophthalmic orbit (33). A conformer implemented during the enucleation surgery is essential to prevent soft tissue contractures within the orbit.

It should be emphasized that the war in Ukraine affects not only soldiers but also civilians including children. Children are the most at-risk population and are often the most severely affected in military conflicts (2). Pediatric ocular trauma is often involved in the posterior segment of the eye, accompanied by multiple tissue damage, irreversible damage to visual function, which brings great difficulty to treatment. The delay of surgical treatment being the consequence of war, makes it more difficult to achieve good anatomical and functional results. The consequences of delay of treatment of eye trauma severely affect children’s visual development and physical and mental health but also cause great loss to family and society. In our case series, two of three members of the same family were 6-year-old children, both of them became monocular after this injury, which means that it will influence their whole life.

In conclusion, cluster munition may lead to bilateral eye trauma, even in the members of the same family and results in multiple IOFBs, close and open eye injuries, and even eye loss. Although it may be treated with modern ophthalmic surgical treatment leading to vision improvement. Thus, to avoid further complications in the postoperative period, pediatric patients after war-related eye trauma should be carefully monitored by an ophthalmologist and a psychologist.

### Patient perspective

An additional psychological consultation was advised, as both children suffered from post-traumatic stress disorder and verbal contact with them was limited. The twins attend a Polish kindergarten and are encouraged to use their vision in everyday activities. The whole family has been staying in Lublin, Poland, in a flat offered by the local authorities and is monitored periodically in the outpatient clinic. In the future, the patients will be further observed for possible delayed complications, as sympathetic ophthalmia, secondary glaucoma, or inflammation.

## Author’s note

We present a case study of one Ukrainian family affected by a claster munition. Three members of the family, including two children, were affected by bilateral eye injuries. Surgical treatment in Poland included cataract surgeries, removal of intraocular foreign bodies, vitrectomy and enucleation. All the members of the family had improved visual acuity and are staying in Poland under supervision. War in Ukraine can result in eye trauma of both adults and children and may lead to severe vision loss if left untreated. Cluster munition may lead to bilateral eye trauma, even in the members of the same family and results in multiply intraocular foreign bodies, close and open eye injuries. Explosion injuries may be treated with modern ophthalmic surgical treatment leading to vision improvement.

## Ethics statement

Written informed consent was obtained from the individual(s), and minor(s)’ legal guardian/next of kin, for the publication of any potentially identifiable images or data included in this article.

## Author contributions

All authors listed have made a substantial, direct, and intellectual contribution to the work and approved it for publication.

## Conflict of interest

The authors declare that the research was conducted in the absence of any commercial or financial relationships that could be construed as a potential conflict of interest.

## Publisher’s note

All claims expressed in this article are solely those of the authors and do not necessarily represent those of their affiliated organizations, or those of the publisher, the editors and the reviewers. Any product that may be evaluated in this article, or claim that may be made by its manufacturer, is not guaranteed or endorsed by the publisher.
